# Idiopathic intracranial hypertension is not benign; a prospective long-term follow-up study

**DOI:** 10.1186/1129-2377-14-S1-P167

**Published:** 2013-02-21

**Authors:** H Yri, M Wegener, B Sander, R Jensen

**Affiliations:** 1 Danish Headache Centre, Department of Neurology, Denmark; 2 Department of Ophthalmology, Glostrup Hospital, University of Copenhagen, Denmark

## Introduction

Idiopathic Intracranial Hypertension (IIH) primarily affects young obese females, and potentially causes visual loss and severe headache.

## Purpose

To examine relapse rate and long-term outcome in patients with IIH.

## Methods

A prospective controlled study of 18 patients with newly diagnosed IIH followed for a mean observation period of 21.1 (± 8.0) months. Treatment regime included diuretics, dietary recommendations and check-up visits at a dietician. Baseline and follow-up included neurological examination, detailed headache history, and comprehensive neuro-ophthalmological examination, including fundus photography, Humphrey visual fields, and measurement of the retinal thickness (RT) and retinal nerve fibre layers (RNFL) by optical coherence tomography (OCT). Relapse was defined as recurrence of either: 1) papilloedema or 2): symptoms and demonstrated raised intracranial pressure.

## Results

Relapse was found in 28%. Visual function improved from baseline to follow-up and was generally favourable. In patients without relapse of papilloedema RT and RNFL were significantly thinner than in healthy controls (p = 0.003 and 0.02), although atrophy was clinically detectable in only one patient. Headache was still present in 67 % of the patients at follow-up. Headache was heterogenic and unrelated to relapse. After an initial weight reduction in both groups, patients in the relapse group gained weight in contrast to patients in the non-relapse group who maintained weight or gained further weight loss (p=0.013).

## Conclusion

Headache was persistent, difficult to classify, and equally represented in relapse and non-relapse patients. Headache was thus a poor marker of active disease. Relapse rate was high, and by OCT we discovered clinically undetectable optic disc atrophy in apparently well treated IIH patients.

